# Cross-sectional analysis of potential risk factors of the pineal gland calcification

**DOI:** 10.1186/s12902-023-01301-w

**Published:** 2023-02-28

**Authors:** Nazanin Jalali, Mohammadrasoul Dehghani Firouzabadi, Ali Mirshekar, Parvin Khalili, Amir reza Ravangard, Jafar Ahmadi, Pooya Saeed Askari, Zahra Jalali

**Affiliations:** 1 Non-Communicable Diseases Research Center, Rafsanjan University of Medical Sciences, Rafsanjan, Iran; 2 Department of Neurology, School of Medicine, Rafsanjan University of Medical Sciences, Rafsanjan, Iran; 3 Student Research Committee, Rafsanjan University of Medical Sciences, Rafsanjan, Iran; 4 Department of Radiology, School of Medicine, Rafsanjan University of Medical Sciences, Rafsanjan, Iran; 5 Social Determinants of Health Research Centre, Rafsanjan University of Medical Sciences, Rafsanjan, Iran; 6 Department of Epidemiology, School of Public Health, Rafsanjan University of Medical Sciences, Rafsanjan, Iran; 7 Student Research Committee, Zabol University of Medical Sciences, Rafsanjan, Iran; 8 Molecular Medicine Research Center, Rafsanjan University of Medical Sciences, Rafsanjan, Iran; 9 Department of Clinical Biochemistry, School of Medicine, Rafsanjan University of Medical Sciences, Rafsanjan, Iran

**Keywords:** Pineal gland, Calcification, Risk factors, Cigarette smoking, Disease history

## Abstract

The Pineal gland (PG) is the site of production of melatonin as an important central hormone in the body. It is not known yet whether PG calcification (PGC) is an age-associated physiological process or a pathologic condition caused by lifestyle-factors and metabolic-dysregulations.

Here, we performed a cross-sectional analysis on 586 patients referred to have Computed Tomographic (CT) scans (above 15 years old), in the Ali Ebne Abi Taleb hospital radiology center in 2017–2018. Based on the CT-scans of the brain, the presence of PGC was recorded and a score of scale 0 to 6 (PGC_score) was calculated for its intensity based on the volume and the Hounsfield units of the calcified pineal. Logistic and ordered logistic regression tests were employed to determine potential risk factor of PGC and higher PGC_score, respectively, testing the factors age, sex, history of cardiovascular and metabolic diseases, smoking and opioid use. We found male sex (OR: 2.30 (95% CI:1.39–3.82) and smoking cigarettes (OR: 4.47 (95% CI:1.01–19.78)) as the main potential risk factors for the pineal gland calcification. For PGC_score, we found age to be dose-dependently associated with PGC_score only in patients aged below 63 (*p*-trend < 0.001). Stratifying for age, in patients < 63 years old, we found age, male sex (positive association) and dyslipidemia (negative association) as the main significantly associated factors of PGC_score. On the contrary, in patients aged >  = 63, cigarette smoking was the only significantly associated factor of higher PGC_score.

In conclusion, our results indicate that at ages below 63, age, male sex and blood lipid are the main associated factors of higher PGC, but at ages above that, the lifestyle factor smoking is significantly associated with higher pineal gland calcification.

**Supplementary Information:**

The online version contains supplementary material available at 10.1186/s12902-023-01301-w.

## Introduction

The pineal gland as the main part of the epithalamus, is known to secrete melatonin as the direct regulator of [1] circadian rhythms in humans [2]. In addition, melatonin has been reported to be involved in neuroprotection against oxidative stress, inflammation, amyloid effects, and apoptosis [3, 4] and its dysregulation has been implicated in several neurodegenerative disorders [3, 5, 6], and stroke [7, 8]. The Pineal gland has a high rate of calcification in the human body forming deposits of magnesium and calcium around corpora arenacea [9]. There have been reports which suggested that the level of melatonin secretion is not affected by pineal calcification and consider this phenomenon as a physiologic process and not associated with aging and disease [10, 11]. On the other hand, some studies have suggested that the pineal gland calcification (PGC) is an age-related pathological process, and the level of 6-sulfatoxymelatonin, the main metabolic form of melatonin, is directly dependent on the size of uncalcified pineal tissue [12, 13].

The chemistry of PGC and the factors which predict the level of pineal calcification are poorly understood and require further studies to determine the main contributing demographic and pathologic factors in PGC induction. There are reports that suggest reduced melatonin levels in smoking and opioid using individuals [14–18]. But studies which investigated smoking and opioid use in relation to the pineal gland calcification are lacking.

Here, we conducted a cross-sectional study on a population of patients referred to Ali Ebne Abi Taleb radiology center in years 2017–2018, assessing the association of PGC and PGC score with the demographic, lifestyle (smoking and opioid use) and history of metabolic and cerebrovascular diseases. To the best of our knowledge this is the first report assessing smoking and opioid use in relation to the pineal gland calcification.

## Methods

### Subjects, study design and ethical considerations

In this cross-sectional analysis, 691 patients (58.4% male, above 15 years old) with a brain CT-scan at the radiology center of the Ali Ebne Abi Taleb hospital at 2017–2018 were reviewed. Patients with low quality CT-scan, pineal tumors or trauma and individuals with incomplete medical records were excluded from our analysis. All procedures of data collection were conducted under the supervision of the Ethics Committee of Rafsanjan University of Medical Sciences (Ethical codes: IR.RUMS.REC.1397.227). The confidentiality of the personal data of participants were ensured by all necessary measures.

### DATA collection and measurements

The archived medical records of the included patients were used to obtain information on their medical history and demographic features including age, sex, history of CVA and current diabetes, hypertension, dyslipidemia, smoking and regular use of opioids (opium, heroin and methadone).

All CT-scans have been performed by SIEMENS machine (SIEMENS Company, Germany) in Axial Plane with a slice thickness of 5 mm without any gap between them. All CT scans were read independently by a neurologist (NJ) and a radiologist (AM)(supplemental Fig. 1). Disagreements were resolved by discussions and consensus. Calcification volume was estimated by measuring length, width and height of the calcified pineal. Patients were categorized to calcified and non-calcified pineal gland groups (PGC). Additionally, they were graded according to the maximal density in Hounsfield units (HU) of the calcified portion of the gland. As suggested by Kunz et al. [19], HU were graded on a five-point Likert scale (0: HU ≤ 49, 1: HU 50–150, 2: 151–250, 3: 251–350, and 4: HU ≥ 351 (HU-Kunz). The calcified volume was categorized to three levels as 0 (if no PGC) and two levels 1 and 2 based on the median volume of calcified pineal glands in the study population. Then, both scores were summed for a total degree of calcification that ranged from 0 to 6 (PGC-score). Categorized PGC_score (cPGC_score) was coded dividing individuals to four groups based on their PGC-score (cPGC_score) as follows: group1:PGC_score 0, group2: PGC_score 1 and 2, group3: PGC_score 3 and 4, group 4: PGC_score 5 and 6. Age was divided to groups by its 10 quantiles as follows: 16–25, 26–34, 35–44, 45–55, 56–62, 63–69, 70–75, 76–80, 81–85, 86–98.

**Fig. 1:**
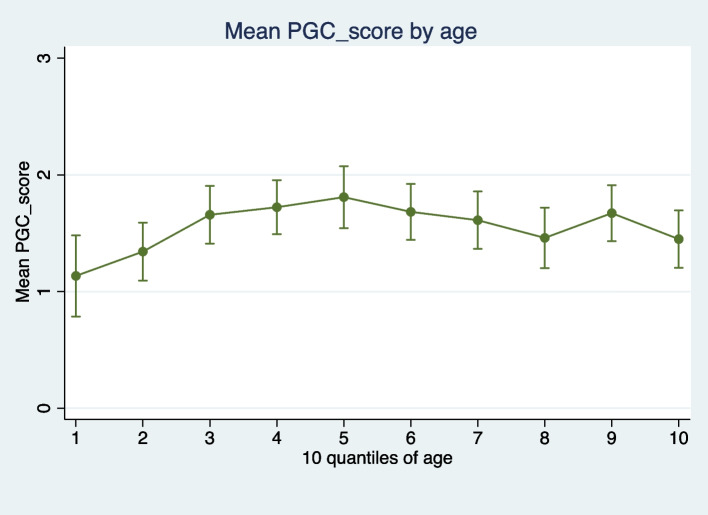
Mean categorized PGC_scores by age group. Data are shown as mean ± 95% Cls

### Statistical analyses

When there was an expected frequency of at least 5 in 80% of the cells, the chi2 test was used for the categorical variables. Otherwise, a Fisher's exact test was used. The normality of the continuous variables was assessed using skewness and kurtosis statistics. All continuous variables in our analysis displayed a normal distribution (age); therefore, independent t-test was used to analyze them. Subject matter knowledge and related epidemiological literature were used for recognition of potential risk factors. In order to find the risk factors in determination of PGC and PGC score, logistic and ordered logistic regression analyses were used respectively at unadjusted and multivariate level to evaluate the potential associated parameters among the following factors: age, sex, cigarette smoking, opioid regular use, history of diabetes Mellitus, hypertension, hyperlipidemia and previous CVA.

Factors which showed *p*-value < 0.2 in the unadjusted analysis, were entered in the respective adjusted logistic or ordered logistic models. The proportional odds ratio assumption was tested with the Brant test. Statistical analyses were performed in Stata software (version 14.1, Stata Corporation, TX, USA). All *p*-values were two-sided. The *p*-values < 0.05 and the 95% confidence intervals not including 1 were considered as statistically significant.

## Results

Table 1 depicts the basic characteristics of the population study. 691 patients were assessed by reading brain CT and medical records. 586 patients were entered in to our analyses after exclusion of patients with low quality CT-scan, pineal tumors or pineal trauma and individuals with incomplete medical records. 84.47% of patients were positive for PGC, and the highest percentage of subjects (28.50%) were calculated a PGC-score of 2 in a scale from 0 to 6. The mean age of PGC (58.90 ± 22.27) and non-PGC cases (55.63 ± 23.28) did not differ significantly (*p*-value = 0.209). The chi^2^ test, displayed a significant association between male sex and PGC (*p*-value < 0.001). Additionally, cigarette smoking and regular opioid use displayed a significant association with PGC (respective *p*-value:0.001, 0.023).

**Table 1:** Baseline characteristics of the study population categorized by PGC

Number (%)	Non PGC	PGC	Total	*p*-value
**Sex (%)**				< 0.001*
Male	37(10.77)	306(89.21)	343 (58.43)	
Female	54(22.13)	190(77.87)	244 (41.57)	
**CVA history**				0.902*
No	66(15.42)	362(84.58)	428 (77.26)	
Yes	20(15.87)	106(84.13)	126 (22.74)	
**Diabetes Mellitus**				0.336*
No	63(14.72)	365(85.28)	428 (77.26)	
Yes	23(18.25)	103(81.75)	126 (222.74)	
**Hyperlipidemia**				0.686*
No	75(15.24)	417(84.76)	492 (88.49)	
Yes	11(17.19)	53(82.81)	64 (11.51)	
**Hypertension**				0.944*
No	54(15.61)	292(84.39)	346 (62.45)	
Yes	32(15.38)	176(84.62)	208 (37.55)	
**Cigarette** **moking**				0.001*
No	78(17.77)	361(82.23)	439 (87.10)	
Yes	2(3.08)	63(96.92)	65 (12.90)	
**Opioid regular use**				0.023*
No	70(17.86)	322(82.14)	392 (77.78	
Yes	10(8.93)	102(91.07)	112 (22.22)	
**PGC-score**				
0	91 (15.53)	0	91 (15.53)	
1	0	19 (3.24)	19 (3.24)	
2	0	167 (28.50)	167 (28.50)	
3	0	125 (21.33)	125 (21.33)	
4	0	76 (12.97)	76 (12.97)	
5	0	58 (9.90)	58 (9.90)	
6	0	50 (8.53)	50 (8.53)	
Mean ± SD				
**Age**	55.63 ± 23.28	58.90 ± 22.27		0.209**

Data are given as Mean ± SD or absolute number n (percentage)

^*^ Chi^2^ test

^**^ Independent t-test

According to the logistic and ordered logistic regression analysis, significant factors related to PGC by univariate logistic regression were found to be male sex, cigarette smoking and regular opioid use. Male sex was associated with more than twice higher odds of PGC (OR: 2.35 (95% CI: 1.49–3.70), *p*-value < 0.001), smoking cigarettes was associated with more than 6 times higher odds ratio of PGC (OR: 6.80 (95% CI: 1.63- 28.40), *p*-value < 0.01), and opioid use was associated with 121% higher odds ratio of PGC (OR: 2.21 (95% CI: 1.10–4.46), *p*-value: 0.02). We next performed multivariate logistic analysis adjusting for factors which displayed an association p-value lower than 0.2 in the unadjusted model. Adjusting for sex, cigarette smoking, and opioid use, we found male sex and smoking cigarettes as the significant risk factors for PGC (male sex adjusted OR: 2.30 (95% CI: 1.39–3.82), *p*-value = 0.001; Cigarette smoking adjusted OR: 4.47 (95% CI: 1.01–19.78), *p*-value = 0.048). We did not find a significant association between opioid use and PGC in the adjusted logistic model (see Table 2).

**Table 2:** Estimated unadjusted and adjusted odds ratios for PGC as predicted by demographic factors and medical history

	**Unadjusted model**		**Adjusted model**	
	**OR (95% CI)**	***p***-value	**OR (95% CI)**	***p*-value**
**PGC**^**#**^				
**Age**	1.00 (0.99–1.01)	0.21		
**Male sex**	2.35 (1.49–3.70)	** < 0.001**	2.30 (1.39–3.82)	**0.001** ^a^
**History of CVA**	0.96 (0.56–1.66)	0.90		
**Diabetes Mellitus**	0.77 (0.45–1.30)	0.33		
**HLP**	0.86 (0.43–1.73)	0.68		
**Hypertension**	1.01 (0.63–1.63)	0.94		
**Cigarette smoking**	6.8 (1.63- 28.40)	** < 0.01**	4.47 (1.01–19.78)	**0.048**^a^
**Opioid use**	2.21 (1.10–4.46)	**0.02**	1.32 (0.62–2.77)	0.46^a^
**Men**				
**Age**	1.00 (0.98–1.01)	0.593		
**History of CVA**	0.63 (0.27–1.44)	0.278		
**Diabetes Mellitus**	0.59 (0.26–1.34)	0.213		
**HLP**	1.60 (0.36–7.09)	0.530		
**Hypertension**	0.83 (0.38–1.80)	0.647		
**Cigarette smoking**	7.44 (0.99–55.98)	**0.051**	7.44 (0.99–55.98)	0.051
**Opioid use**	1.67 (0.65–4.26)	0.283		
**Women**				
**Age**	1.02 (1.00–1.03)	**0.012**	1.02 (1.00–1.036)	**0.012**
**History of CVA**	1.48 (0.71–3.11)	0.293		
**Diabetes Mellitus**	1.08 (0.54–2.16)	0.818		
**HLP**	0.080 (0.35–1.85)	0.617		
**Hypertension**	1.54 (0.82–2.86)	0.173		
**Cigarette smoking**	2.84 (0.35–23.03)	0.326		
**Opioid use**	1.97 (0.65–5.97)	0.230		
**Categorized PGC score**^**##**^				
**Age**	1.00 (0.99–1.01)	0.21		
**Male sex**	2.34 (1.48–3.69)	** < 0.001**	1.72 (1.23–2.39)	**0.001** ^b^
**History of CVA**	0.91 (0.63–1.30)	0.617		
**Diabetes Mellitus**	1.21 (0.84–1.74)	0.300		
**HLP**	0.87 (0.54–1.39)	0.566		
**Hypertension**	0.940 (0.689–1.28)	0.698		
**Cigarette smoking**	1.84 (1.15- 2.93)	**0.010**	1.56 (0.97–2.51)	**0.062**^b^
**Opioid use**	1.24 (0.85–1.81)	0.247		
**Men**				
**Age**	1.00 (0.99–1.01)	0.095	1.00 (0.99–1.01)	0.702^c^
**History of CVA**	0.78 (0.47–1.28)	0.335		
**Diabetes Mellitus**	1.05 (0.62- 1.75)	0.852		
**HLP**	0.85 (0.43–1.67)	0.649		
**Hypertension**	1.07 (0.68–1.66)	0.763		
**Cigarette smoking**	1.85 (1.088–3.16)	**0.023**	1.76 (1.02–3.02)	**0.039**^c^
**Opioid use**	1.17(0.74- 1.86)	0.484		
**Women**				
**Age**	1.00 (0.99- 1.01)	0.263		
**History of CVA**	1.19(0.71–2.01)	0.495		
**Diabetes Mellitus**	1.61 (0.95–2.72)	0.075	1.61 (0.95–2.72)	0.075
**HLP**	0.99 (0.51- 1.92)	0.995		
**Hypertension**	1.05 (0.66–1.67)	0.804		
**Cigarette smoking**	0.88 (0.30–2.53)	0.814		
**Opioid use**	1.958(0.48–1.90)	0.905		
**Age < 63**				
**Male sex**	1.69 (1.09–2.62)	**0.017**	2.82 (1.72–4.62)	** < 0.001**^**d**^
**Age**	1.02 (1.00–1.03)	**0.002**	1.03 (1.01–1.05)	** < 0.001**^**d**^
**History of CVA**	1.38 (0.70–2.72)	0.34		
**Diabetes Mellitus**	1.13 (0.60–2.13)	0.68		
**HLP**	0.44 (0.17–1.11)	0.083	0.33 (0.13–0.87)	**0.025**^**d**^
**Hypertension**	1.30 (0.78–2.16)	0.309		
**Cigarette smoking**	1.24 (0.62–2.46)	0.535		
**Opioid use**	1.32 (0.75–2.32)	0.33		
**Age > = 63**				
**Male sex**	1.72 (1.13–2.62)	**0.011**	1.39 (0.88–2.19)	0.152^e^
**Age**	0.99 (0.96–1.01)	0.42		
**History of CVA**	0.74 (0.47–1.16)	0.190		
**Diabetes Mellitus**	1.26 (0.79–2.00)	0.32		
**HLP**	1.11 (0.63–1.94)	0.703		
**Hypertension**	0.73 (0.47–1.11)	0.145		
**Cigarette smoking**	2.57 (1.36–4.85)	**0.003**	2.31 (1.20–4.42)	**0.011**^**e**^
**Opioid use**	1.18 (0.71–1.96)	0.504		
**Cigarette smoking (in men only)**	2.94 (1.40–6.18)	**0.004**		
**Cigarette smoking (in women only)**	1.05 (0.26–4.23)	0.94		

^#^logistic regression analysis

^##^Ordered logistic regression analysis

^a^ Adjusted for sex, cigarette smoking and regular opioid use

^b^ Adjusted for sex and cigarette smoking

^c^ Adjusted for age and cigarette smoking

^d^ Adjusted for age decile, sex and hyperlipidemia

^e^ Adjusted for sex and cigarette smoking

We next used sensitivity analysis by sex-stratification to investigate gender-specific associations of PGC with different factors. In men an odds ratio of 7.44 (95% CI: 0.99–55.985), *p*-value = 0.051) was observed for PGC in association with cigarette smoking. In female subjects age displayed a significant association with PGC (OR: 1.02 (95% CI: 1.00–1.036), *p*-value = 0.012).

Performing ordered logistic regression analysis on the potential risk factors for the categorized PGC-score (cPGC_score), in the unadjusted model, male sex and cigarette smoking displayed a statistically significant association with cPGC_score (respective *p*-value: < 0.001, 0.01). In the multivariate ordered logistic test, male sex showed a significant association with cPGC_score (adjusted OR: 1.72 (95% CI: 1.23–2.39), *p*-value: 0.001). In addition to adjusting for gender, we performed a sensitivity analysis by sex-stratification. In the unadjusted ordered logistic model in men cigarette smoking displayed a significant association with cPGC_score (*p*-value:0.23). Also, in the multivariate analysis in men only, cigarette smoking was found to be significantly associated with higher cPGC_score (adjusted OR: 1.76 (95% CI: 1.02–3.02), *p*-value: 0.039). Performing the same analysis in women, we did not find a statistically significant association between cigarette smoking and higher cPGC-score (adjusted OR: 0.88 (95% CI: 0.30–2.53), *p*-value: 0.814).

Figure 1 indicates the mean categorized PGC_scores for the 10 quantiles of the age. Based on this graph, until the 5^th^ decile of age (below 63 years old in our population), there was an increasing trend of PGC_score by age. Therefore, we added ordered logistic analysis stratified by age (below age 63 and above 63) for different potential risk factors (Table 2). Our results indicated that in patients aged < 63 years: age (adjusted OR: 1.03 (95% CI: 1.01–1.05), *p*-value < 0.001) and male sex (adjusted OR: 2.82 (95% CI: 1.72–4.62), *p*-value < 0.001) were the two main positive associated factors of higher PGC_score, and hyperlipidemia (adjusted OR: 0.33 (95% CI: 0.13–0.87), *p*-value = 0.025) was the main factor negatively associated with higher PGC_score. A dose–response linear trend was observed for the categorized PGC_score and age deciles below 63 (*p*-trend < 0.001) (Table 3). On the contrary, in the patients aged 63 and above, the only significant associated factor of PGC_score was found to be cigarette smoking (adjusted OR: 2.31(95% CI: 1.20–4.42), *p*-value = 0.011). Additionally, when performed separated analysis for men and women, we found that the association of cigarette smoking and PGC_score in patients aged 63 and above, is only significant in male objects (OR: 2.94 (95% CI: 1.40–6.18), *p*-value: 0.004). The gender differential results may be probably driven from residual confounding from gender or its interaction effects with smoking. Additionally, the reason may be the small number of smoking women compared to men (supplemental Table 1).

**Table 3:** Estimated unadjusted and adjusted odds ratios for categorized PGC_score as predicted by 10 quantiles of age

	**Unadjusted Model**		**Adjusted Model**	
	**OR (95% CI)**	***p*-value**	**OR (95% CI)**	***p*-value**
**Categorized PGC_score##**				
**Age < 63 years old**				Linear *p*-Trend < 0.001^a^
1^st^ decile (16–25)	Reference		Reference	
2^nd^ decile (26–34)	1.19 (0.62–2.26)	0.588	1.49(0.75–2.93)	0.247
3^rd^ decile (35–44)	2.00 (1.04–3.85)	0.035	2.79 (1.36–5.72)	0.005
4^th^ decile (45–55)	2.17 (1.15- 4.10)	0.016	3.30 (1.62–6.70)	0.001
5^th^ decile (56–62)	2.17 (1.14–4.17)	0.018	3.59 (1.74–7.40)	0.001

^##^Ordered logistic regression analysis

^a^ Adjusted for age_decile, sex and hyperlipidemia

## Discussion

We performed a cross-sectional study to investigate the association of demographic and personal habits with the pineal gland calcification. We found that the male sex is one of the factors significantly associated with PGC. Future studies are required to investigate the underlying reason for this gender difference in risk of pineal calcification.

In addition to male sex, our adjusted logistic analysis suggests cigarette smoking as a potent factor associated with increased odds of PGC (OR: 4.47 (95% CI: 1.01–19.78), cPGC_score in men (OR: 1.76 (95% CI: 1.02–3.02)), and cPGC_score in patients >  = 63 years (OR: 2.31(95% CI: 1.20–4.42)). We did not find any association between opioid regular use and PGC in the adjusted regression analyses. To the best of our knowledge, no previous study has assessed the connection of smoking and opioid use with PGC which is a unique character and strength of the present study. There are previously published evidences that support a link between smoking and opioid use with decreased melatonin levels [14–18]. Our results do not indicate opioid use effect on melatonin to occur through inducing PGC. Previous studies showing the effect of smoking on melatonin levels, have indicated changes in the pharmacokinetic parameters of melatonin such as a lowered C_max_ (serum maximum concentration) when exogenous melatonin was injected. This study showed a pharmacokinetic effect of smoking for removal of melatonin from the body as the underlying mechanism for this effect, and suggested an impact of smoking independent of the pineal gland activity [20]. Our results showed an association between smoking and the pineal gland calcification. We propose future studies to investigate whether the decreasing effect of smoking on melatonin levels may be mediated at least partially by increasing the pineal gland calcification.

Smoking has been shown by several previous studies to be a risk factor for vascular calcification in different tissues [21–24]. The suggested underlying mechanisms are the smoking-induced oxidative stress and alterations in the vesicular trafficking in the vascular smooth muscle cells [20]. Future studies are required to ask whether smoking may induce pineal gland calcification via similar mechanisms.

There has been variation in the results of the former reports assessing whether the pineal gland calcification is a function of age or not. Some previous studies support a direct association between aging and PGC in all ages in human and animal studies [25, 26], proposing PGC is an inevitable process of aging; while some other studies found that the increase in PGC by age is observed only by certain age (60 years old), and above this age the correlation disappears or is reversed [27]). Our results conform to the later, showing an age-dependent increase in PGC_score only in patients aged below 63. In younger individuals, previous studies have shown that PGC is not observed before age 5, but shows an age-dependent increase from age older than 5 to 20 years old [28, 29]. Here, we have assessed PGC in patients above 15 years old, and we observed a significant association between PGC and age only in patients younger than 63 years old.

Given that the gold standard method in diagnosing the pineal gland calcifications (PGC) can only be achieved by postmortem investigation of the pineal gland, we propose future anatomo-histological studies on postmortem biopsy samples to assess the association of smoking and PGC. Previous post mortem pineal gland studies found pineal calcification at the highest rate in the age group of 46–65 years old, but no differences between genders were observed [30–32].

Some previous studies have suggested calcification as a commonly dominant feature of cystic pineal glands [33–39]. Future studies are required to assess whether there is a relationship between the risk of pineal gland calcification and cysts.

One limitation of our study is the lack of information on some of the potential risk factors of the pineal gland calcification, such as the body mass index, alcohol consumption, diet, physical activity and sunlight exposure. However, a complete medical history record for each of patients was available providing valuable information on the current diseases of the patients including diabetes mellitus, hyperlipidemia, hypertension and cerebrovascular diseases. The other limitation of the present study is the lack of information on the start age of the above-mentioned diseases or duration of smoking and opioid addiction or the dosage of their use.

In conclusion, we found that until age 63, age and male sex are the two potential associated risk factors for pineal gland calcification, and above this age smoking cigarettes may be a risk factor for PGC, which warrants further investigation in the future.

## Supplementary Information

**Additional file 1.**

**Additional file 2. **

## Data Availability

The datasets generated and analyzed during the current study are not publicly available but are available from the corresponding author on reasonable request.
