# Supplementary material for: Cross-sectional analysis of potential risk factors of the pineal gland calcification
Source: BMC Endocr Disord. 2023 Feb 28;23:49. doi: 10.1186/s12902-023-01301-w (PMC9972749; doi:10.1186/s12902-023-01301-w)
Supplement: Supplementary file 1 — Additional file 1. [file 12902_2023_1301_MOESM1_ESM.docx]

| Supplemental table 1. habits of the study population categorized by sex | | | | |
| --- | --- | --- | --- | --- |
| Number (%) | **Male** | **Female** | **Total** | **p-value** |
| Cigarette smoking |  |  |  | <0.001 |
| Non-smoker | 231(80.77) | 208 (95.41) | 439 (87.10) |  |
| smoker | 55(19.23) | 10 (4.59) | 65 (12.9) |  |
| Opioid use |  |  |  | <0.001 |
| non-user | 202 (70.63) | 190 (87.16) | 392 (77.78) |  |
| user | 84 (29.37 ) | 28 (12.84) | 112 (22.22) |  |

Data are given as Mean±SD or absolute number n (percentage)
