# Supplementary material for: Cross-sectional analysis of potential risk factors of the pineal gland calcification
Source: BMC Endocr Disord. 2023 Feb 28;23:49. doi: 10.1186/s12902-023-01301-w (PMC9972749; doi:10.1186/s12902-023-01301-w)
Supplement: Supplementary file 2 — Additional file 2. [file 12902_2023_1301_MOESM2_ESM.zip › supp. figure 1.pdf]

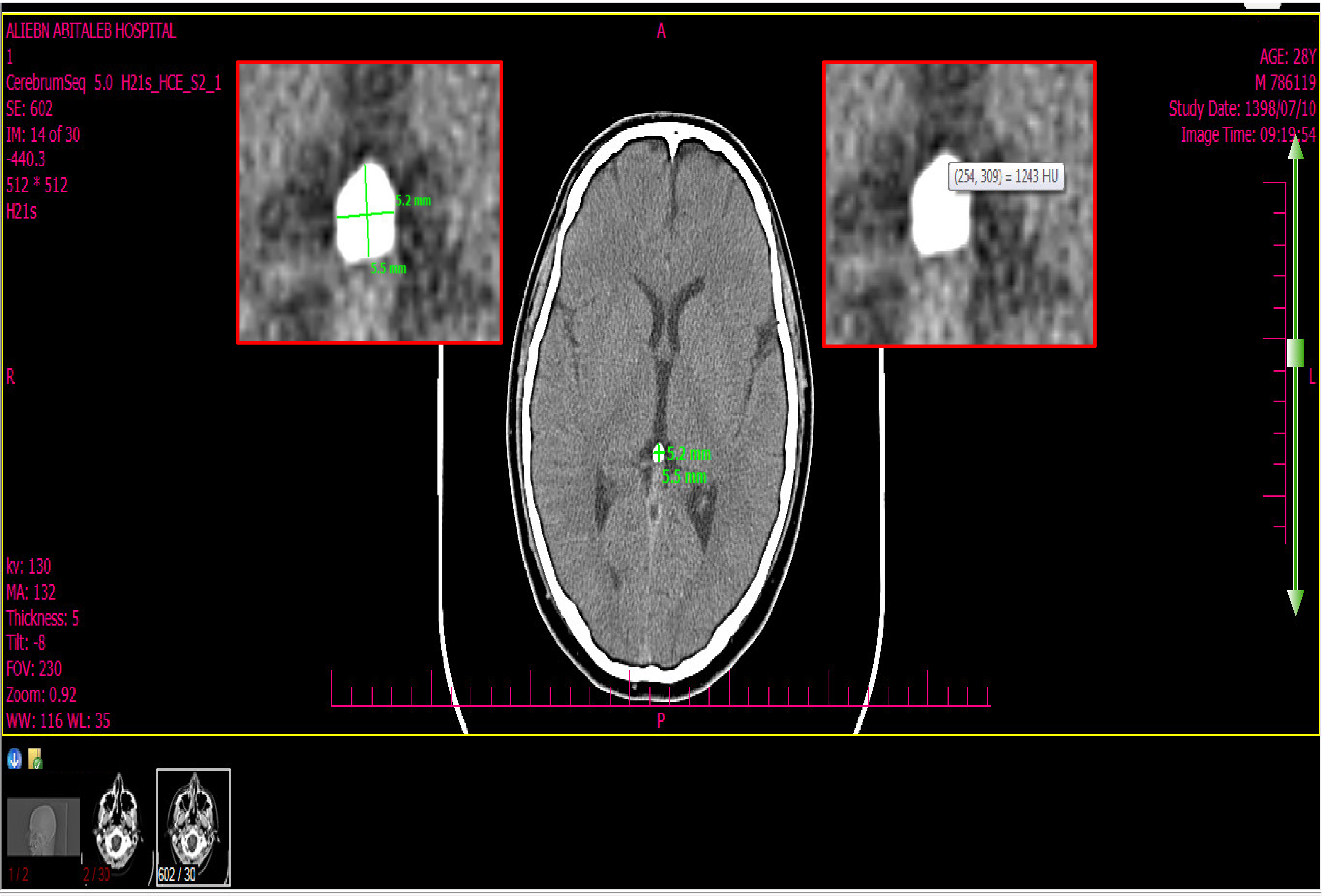
ALIEBN ABITALEB HOSPITAL
1
CerebrumSeq 5.0 H21s_HCE_S2_1
SE: 602
IM: 14 of 30
-440.3
512 * 512
H21s
A
AGE: 28Y
M 786119
Study Date: 1398/07/10
Image Time: 09:19:54
5.2 mm
5.5 mm
(254, 309) = 1243 HU
R
L
kv: 130
MA: 132
Thickness: 5
Tilt: -8
FOV: 230
Zoom: 0.92
WW: 116 WL: 35
P
1 / 2
2 / 30
602 / 30

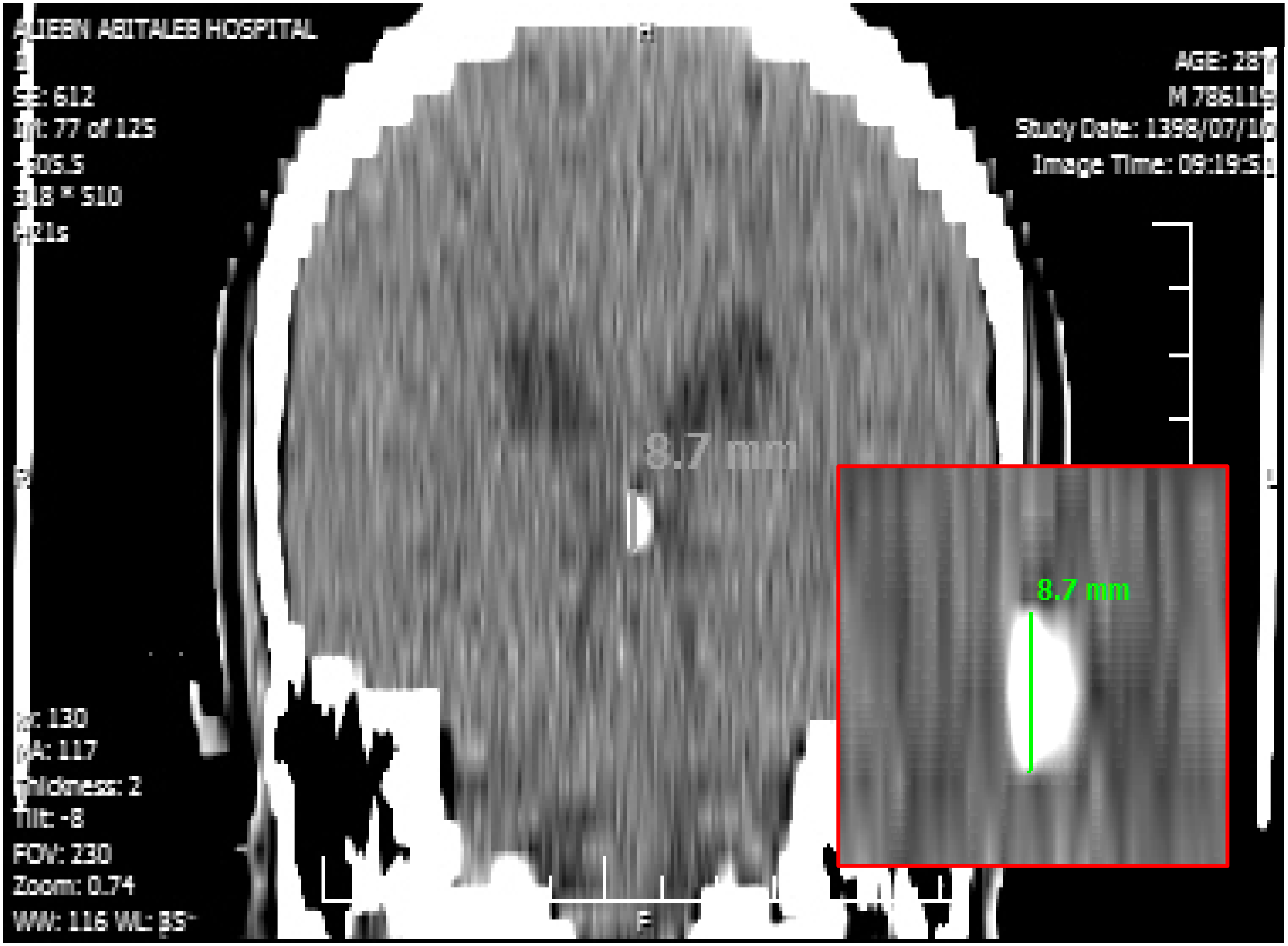

Supplemental figure 1. CT-scan images of the brain of a representative patient
a. The measurements of the length and the width diameters of the pineal gland calcification and HU b. The measurement of depth of the pineal gland calcification.
